# CT-to-PET Synthesis in the Head–Neck and Thoracic Region via Conditional 3D Latent Diffusion Modeling

**DOI:** 10.3390/bioengineering13050534

**Published:** 2026-05-03

**Authors:** Mohammed A. Mahdi, Mohammed Al-Shalabi, Reda Elbarougy, Ehab T. Alnfrawy, Muhammad Usman Hadi, Rao Faizan Ali

**Affiliations:** 1 Information and Computer Science Department, College of Computer Science and Engineering, University of Ha’il, Ha’il 55476, Saudi Arabia; m.mahdi@uoh.edu.sa (M.A.M.); moh.alshalbi@uoh.edu.sa (M.A.-S.); 2 Artificial Intelligence and Data Science Department, College of Computer Science and Engineering, University of Ha’il, Ha’il 55476, Saudi Arabia; reda.elsayed@uoh.edu.sa; 3 Information Security Department, College of Computer Science and Engineering, University of Ha’il, Ha’il 55476, Saudi Arabia; eha.alnfrawy@uoh.edu.sa; 4 School of Engineering, Ulster University, Belfast BT15 1AP, UK; m.hadi@ulster.ac.uk; 5 School of Computing, University of Kent, Canterbury CT2 7NZ, UK

**Keywords:** CT-to-PET translation, latent diffusion model, conditional generative modeling, 3D medical image synthesis, PET reconstruction, SUV-based evaluation

## Abstract

**Background**: Positron emission tomography (PET) provides physiologic information central to oncologic staging and treatment assessment, but its availability is limited by cost, radiation exposure, and scanner access. Synthesizing PET from computed tomography (CT) is attractive but challenging, as tracer uptake is only partially constrained by anatomy, making the mapping inherently one-to-many. **Methods**: We propose a conditional 3D latent diffusion framework (3D-LDM) for CT-to-PET synthesis in the head–neck and thoracic region. The pipeline localizes anatomy by segmenting lungs in CT and restricting the volume to reduce irrelevant variability. PET volumes are encoded into a compact latent space using a KL-regularized 3D autoencoder, and a conditional 3D diffusion U-Net learns to generate PET latents conditioned on CT via a denoising diffusion process. The model was trained and evaluated on 900 paired PET/CT studies. Performance was assessed in SUV space using MAE, PSNR, and SSIM, and compared against transformer-, CNN-, and GAN-based baselines. **Results**: On the held-out test cohort, 3D-LDM achieved the best overall quantitative fidelity (MAE = 303.05 ± 22.16 SUV units, PSNR = 32.64 ± 1.79, SSIM = 0.86 ± 0.03), outperforming all baselines with statistically significant differences (*p* < 0.001). At the lesion level, the model achieved a precision of 0.76 (95% CI: 0.71, 0.81) and recall of 0.76 (95% CI: 0.72, 0.80), detecting an average of 3.19 lesions per scan with a false-positive rate of 0.72/scan. Lesion-wise NMSE was 11.37%, significantly outperforming GAN and transformer baselines. **Conclusions**: 3D-LDM enables efficient, high-fidelity PET synthesis in the head–neck and thoracic regions, substantially improving lesion-level accuracy over state-of-the-art baselines. While it is not a replacement for diagnostic PET, these results support the model’s potential as a clinical decision support tool.

## 1. Introduction

Positron emission tomography/computed tomography (PET/CT) has become a cornerstone of modern oncologic imaging because it combines high-resolution anatomy from CT with functional and molecular information from PET [1,2,3,4], enabling staging [5,6], treatment planning [7], and response assessment that cannot be achieved reliably with anatomy alone [8,9]. Nevertheless, PET remains comparatively costly and resource-intensive [10], introduces additional ionizing radiation exposure, and is not uniformly accessible across health systems, creating a persistent gap between the clinical value of PET and the real-world feasibility of deploying it at scale [11,12]. These limitations motivate the development of data-driven approaches that can estimate PET-like metabolic information from widely available CT acquisitions, with the long-term goal of reducing barriers to quantitative molecular imaging and supporting more consistent diagnostic workflows [13,14].

CT-to-PET translation, however, is intrinsically challenging [1,2]. CT encodes tissue density and morphology, whereas PET uptake reflects complex physiology (e.g., glucose metabolism, inflammation, tumor microenvironment, and therapy effects) that is only partially constrained by anatomy [15]. As a result, the mapping from CT to PET is fundamentally one-to-many: multiple plausible uptake patterns may be compatible with the same CT appearance, especially for subtle lesions and physiologic tracer activity [1,12]. This ambiguity imposes stringent accuracy requirements for clinical applicability, because PET is interpreted quantitatively and even modest scaling errors may influence staging, therapy monitoring, and longitudinal comparison. Many earlier deep learning (DL) approaches for cross-modality synthesis rely on deterministic regressors or adversarial training, which can struggle under one-to-many ambiguity and may produce over-smoothed outputs, biased SUV scaling, or structured artifacts that are especially consequential in diagnostic and quantitative settings [16,17,18,19,20,21,22]. Moreover, uptake patterns often extend across multiple slices (e.g., nodal chains and multi-focal disease), motivating true volumetric modeling; however, full-resolution 3D generation in voxel space can be computationally prohibitive [23,24].

Diffusion models have recently emerged as a powerful class of generative models that naturally fit one-to-many mappings by learning a conditional distribution rather than a single point estimate [25]. In particular, latent diffusion models (LDMs) perform the diffusion process in a compressed latent space, substantially improving computational efficiency while retaining high-fidelity synthesis capabilities relative to pixel-space diffusion [26]. This combination of probabilistic generation plus efficiency makes conditional latent diffusion especially attractive for 3D medical imaging problems where memory and compute constraints have historically limited model capacity and volumetric context [27,28,29,30].

In this work, we propose a Conditional 3D Latent Diffusion (3DLDM) for CT-to-PET translation, designed to capture volumetric context while remaining computationally tractable through latent-space diffusion. Building on the probabilistic nature of diffusion, our approach aims to model the inherent uncertainty of metabolic uptake conditioned on anatomy, rather than forcing a single deterministic reconstruction. We develop and evaluate the model using 900 patients, leveraging the paired whole-body FDG-PET/CT imaging. By focusing on 3D consistency and distributional modeling, our goal is to produce synthetic PET volumes that are anatomically aligned, quantitatively faithful enough to support downstream analysis, and robust across diverse lesion presentations, providing a practical step toward scalable PET-informed imaging in settings where PET acquisition is limited.

The remainder of this paper is organized as follows. Section 2 reviews related work. Section 3 describes the dataset, preprocessing, the proposed conditional 3D latent diffusion model, and the experimental and evaluation protocols. Section 4 reports quantitative results and representative qualitative examples highlighting strengths and failure modes. Section 5 discusses clinical implications, limitations, and safety/ethical considerations. Section 6 concludes and outlines future directions to improve fidelity, generalization, and downstream utility in oncologic PET/CT workflows.

## 2. Related Work

Cross-modality synthesis has long been investigated as a way to reduce scan burden, mitigate missing modalities, and enable downstream tasks that require paired information (e.g., attenuation correction, target delineation, or therapy planning) [31]. Early cross-modality synthesis pipelines were dominated by encoder–decoder CNNs (often U-Net-like architectures) that learn paired mappings with strong spatial inductive bias, and by conditional Generative Adversarial Network (GAN) frameworks that improve perceptual realism via adversarial training (e.g., pix2pix) [32]. Because perfectly paired or perfectly aligned medical data are not always available, unpaired translation strategies such as CycleGAN also became influential, especially when registration noise or protocol variability makes one-to-one supervision imperfect [33]. Despite their success, these approaches often regress toward an “average” solution under ambiguous mappings and can exhibit instability or hallucinated structures, failure modes that are particularly concerning when the synthesized modality is used for oncology decision support.

CT-to-PET (“virtual PET”) translation is substantially harder than anatomy-to-anatomy translation because PET uptake reflects biology and physiology that are only partially constrained by anatomy, making the problem inherently one-to-many. Early work demonstrated feasibility using Fully Convolutional Networks (FCNs) and conditional GANs to estimate PET from CT in limited cohorts (often constrained anatomically, e.g., liver-focused settings), providing important proof-of-concept but also highlighting the dependency on dataset size, disease distribution, and acquisition consistency [21,31,34,35]. More broadly, surveys of medical translation methods, covering synthetic PET among other targets, emphasize recurring challenges: cross-site generalization, quantification fidelity, and evaluation practices that align with downstream clinical tasks rather than only pixel-wise similarity [16]. In parallel, community benchmarks such as the autoPET challenge have provided large-scale, annotated oncologic FDG-PET/CT datasets and standardized evaluation that clarify what it takes to perform robustly across heterogeneous whole-body scans, thereby offering a stronger substrate for learning and validating PET-related image generation [15].

Diffusion models have recently shifted the landscape because they model conditional distributions more naturally than deterministic regressors or GANs, which is attractive for one-to-many mappings like CT-to-PET. Denoising diffusion probabilistic models (DDPMs) and score-based formulations provide stable training objectives and strong mode coverage at the cost of iterative sampling [36]. To address computational constraints, which are especially acute in 3D medical imaging, latent diffusion models (LDMs) perform the diffusion process in a learned compressed latent space, substantially improving efficiency while preserving high-frequency detail via decoding [26]. Medical imaging-focused reviews document rapid adoption of diffusion models across synthesis and reconstruction tasks, but also stress practical issues such as computation issues, validation rigor, and safety [37]. Volumetric extensions that explicitly target 3D consistency (e.g., latent diffusion-based paradigms for 3D cross-modality synthesis) further motivate 3D diffusion designs for whole-body imaging [38]. Importantly, diffusion specifically for CT-to-PET is now emerging; recent work has proposed diffusion-based CT-to-PET translation (e.g., CPDM) and introduced larger-scale datasets and protocols, suggesting clear momentum while leaving room for improved 3D conditioning, efficiency, and clinically meaningful evaluation on standardized benchmarks [39].

## 3. Methods

Our framework employs a two-stage 3D latent diffusion model to synthesize high-quality PET volumes from CT input, operating in a compressed latent space to reduce computational overhead while retaining clinically relevant structural and uptake fidelity.

### 3.1. Dataset

This study was conducted using paired whole-body oncologic PET/CT data from the publicly available autoPET II [15], which provides FDG-PET/CT imaging with expert lesion annotations to support benchmarking in oncologic imaging. We have used 900 patients and partitioned them at the patient level into 600 training, 150 validation, and 150 testing cases to avoid information leakage across splits. Each case contains a CT volume acquired as part of the PET/CT protocol and a corresponding PET reconstruction from the same session, enabling paired learning. The goal is to synthesize PET from CT within a clinically motivated head–neck and thoracic field-of-view.

Importantly, the cohort includes examinations from two medical centers (University Hospital Tübingen and LMU University Hospital Munich) and was acquired on multiple PET/CT systems spanning different vendors, including Siemens Biograph-series scanners (Siemens Healthineers AG, Forchheim, Germany) and GE Discovery 690 (General Electric Company, Chicago, IL, USA). Acquisition and reconstruction settings vary across sites (e.g., injected activity, CT parameters, slice thickness, and PET reconstruction), providing intrinsic protocol heterogeneity that better reflects real-world variability and supports a more realistic assessment of cross-protocol robustness.

### 3.2. Data Preprocessing

To reduce inter-scan variability and to focus the model on clinically relevant uptake patterns in the head–neck and thoracic region, we applied a standardized preprocessing pipeline. First, all CT and PET volumes were converted to a common orientation (RAS) and resampled to a consistent voxel spacing to ensure spatial compatibility across patients. Second, CT intensities were clipped to suppress outliers and then normalized to a fixed range to stabilize training. PET intensities were normalized to standardized uptake value (SUV) units, as provided by the autoPET II dataset, and clipped to limit extreme values from physiological uptake hotspots.

Critically, we restricted the field of view using an anatomy-guided crop derived from CT lung segmentation. We generated lung masks on CT using the open-source lungmask package (v0.2.13) (pretrained U-Net models for automated lung segmentation) [40] and used the resulting masks to identify the inferior lung extent. We then cropped each paired CT/PET volume from the lung base superiorly to the head, thereby excluding abdominal and pelvic regions and ensuring that the learning problem is concentrated on thoracic structures and head–neck tissues where metastases and clinically meaningful uptake patterns are frequently assessed. This crop also reduces memory footprint and mitigates confounding from strong physiological abdominal uptake. After lung-to-head cropping, we applied a fixed-size crop (and padding when needed) to obtain a uniform 3D input size for training, which ensures a computationally tractable representation while preserving volumetric context.

### 3.3. Problem Formulation

Given a preprocessed CT volume x within the head–neck and thoracic field-of-view, the objective is to generate a synthetic PET volume $\hat{y}$ in SUV units that matches the distribution of the corresponding ground-truth PET y. Because metabolic uptake is not uniquely determined by anatomy, the mapping is inherently one-to-many; thus, we model the conditional distribution p(y∣x) with a conditional latent diffusion model rather than a deterministic regressor.

### 3.4. Overview of the Proposed Conditional 3D Latent Diffusion Framework

**Figure 1:**
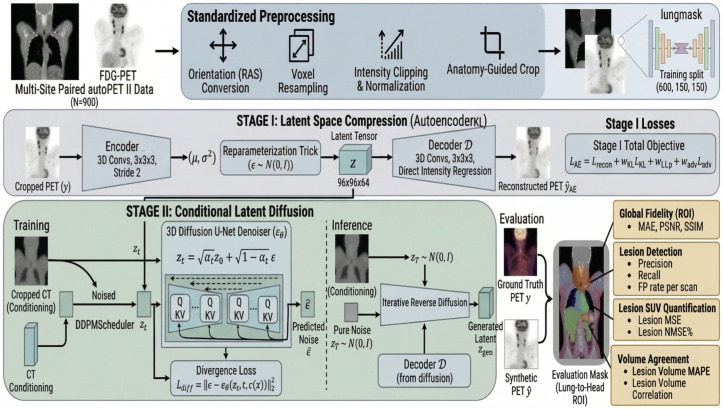
Overview of the proposed conditional 3D latent diffusion (3D-LDM) for CT-to-PET translation in the head–neck and thoracic region.

**Figure 2:**
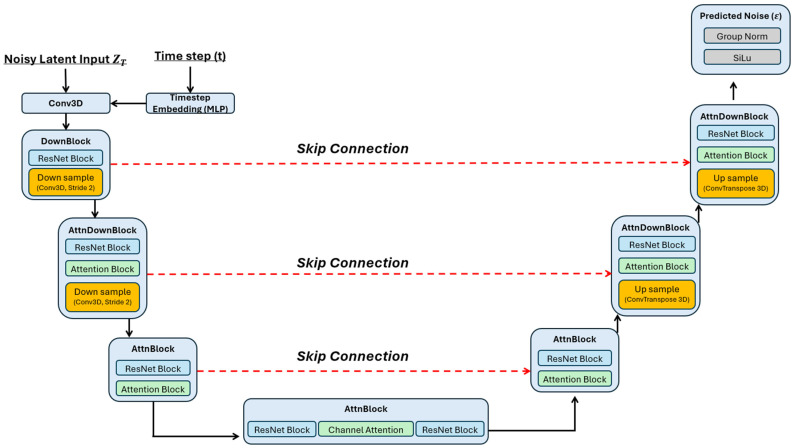
Architecture of the 3D diffusion U-Net denoiser used in latent space for conditional CT-to-PET generation.

We propose a Conditional 3D Latent Diffusion (3DLDM) for CT-to-PET translation in the head–neck and thoracic region. The method follows a two-stage strategy typical of latent diffusion: (i) a variational autoencoder learns a compact latent representation of PET volumes, and (ii) a 3D diffusion U-Net learns to generate PET latents conditioned on CT. Performing diffusion in latent space substantially reduces computational cost compared with voxel-space diffusion while maintaining high-fidelity synthesis. The overall workflow is illustrated in Figure 1, and the denoising network architecture is detailed in Figure 2.

#### 3.4.1. Stage I: Latent Space Compression via AutoencoderKL

To facilitate computationally efficient diffusion training, we employ a Variational Autoencoder (AutoencoderKL) to map the high-dimensional PET target volume y into a low-dimensional latent embedding z. The encoder E parameterizes the latent distribution by predicting the mean μ and variance $\sigma^{2}$. The latent representation is modeled as a Gaussian distribution:

(1) $$q(z|y)=N\left(z;\mu (y),diag\left(\sigma^{2}(y)\right)\right),$$

To allow for backpropagation, a latent sample z is drawn using the reparameterization trick:

(2) z=μ(y)+σ(y)⊙ϵ, where ϵ∼N(0,I),

The decoder D subsequently reconstructs the PET volume from this latent code such that $\hat{y}=D(z)$. To ensure the latent space captures high-frequency details essential for medical diagnosis, the Autoencoder is optimized using a composite objective function consisting of a voxel-wise reconstruction loss ($L_{\mathrm{recon}}$), a KL-divergence regularization term ($L_{KL}$), a perceptual loss $\left(L_{p}\right)$, and an adversarial loss $\left(L_{\mathrm{adv}}\right)$. The total objective for the first stage is formulated as:

(3) $$L_{AE}=L_{\mathrm{recon}}+w_{KL}L_{KL}+w_{p}L_{p}+w_{\mathrm{adv}}L_{\mathrm{adv}}$$

where $L_{\mathrm{recon}}=\|y-D(E(y)){\|}_{1}$ enforces L1 pixel-level consistency, and $L_{KL}=\frac{1}{2}\sum \left(\mu_{i}^{2}+\sigma_{i}^{2}-log\left(\sigma_{i}^{2}\right)-1\right)$ aligns the latent distribution with a unit Gaussian to prevent overfitting. Each PET volume of size 96×96×64 is compressed into a latent tensor with 3 channels. All 3D convolutions use 3×3×3 kernels; downsampling is implemented with stride 2, while other convolutions use stride 1. We use SiLU (Swish) activations in intermediate layers and omit the final activation in the decoder output layer to allow direct regression of PET intensity (SUV) values.

#### 3.4.2. Stage II: Conditional Latent Diffusion

The core generative process is handled by a DiffusionModelUNet operating in the learned latent space. This network learns to reverse a gradual noising process, generating valid PET latent representations conditioned on anatomical CT priors. Given a clean latent code $z_{0}$ (encoded from the ground truth PET), the forward process iteratively corrupts the data with Gaussian noise over T timesteps. The transition kernel is defined as a Markov chain:

(4) $$q\left(z_{t}|z_{t-1}\right)=N\left(z_{t};\sqrt{\alpha_{t}}z_{t-1},\left(1-\alpha_{t}\right)I\right),$$

where $\alpha_{t}=1-\beta_{t}$ represents the noise schedule. Utilizing the property ${\bar{\alpha }}_{t}=\prod_{s=1}^{t}\alpha_{s}$, we can sample $z_{t}$ at any arbitrary timestep directly:

(5) $$z_{t}=\sqrt{{\bar{\alpha }}_{t}}z_{0}+\sqrt{1-{\bar{\alpha }}_{t}}\epsilon ,\epsilon ∼N(0,I),$$

We employ a noise-prediction network $\epsilon_{\theta }\left(z_{t},t,c(x)\right)$, where c(x) represents the CT-based conditioning is injected via channel-wise concatenation mechanisms. The model is trained to predict the added noise ϵ by minimizing the simple mean squared error:

(6) $$L_{diff}={\left\|\epsilon -\epsilon_{\theta }\left(z_{t},t,c(x)\right)\right\|}_{2}^{2},$$

During inference, we generate a synthetic PET volume by starting from pure Gaussian noise $z_{T}∼N(0,I)$ and iteratively applying the reverse diffusion step from t=T to 1:

(7) $$z_{t-1}=\frac{1}{\sqrt{\alpha_{t}}}\left(z_{t}-\frac{1-\alpha_{t}}{\sqrt{1-{\bar{\alpha }}_{t}}}\epsilon_{\theta }\left(z_{t},t,c(x)\right)\right)$$

### 3.5. Experimental Configuration and Reproducibility

To ensure the reproducibility of our results, we implemented the proposed 3DLDM framework using PyTorch (v.2.0.0) and MONAI (v.1.3.0) on NVIDIA A100 GPUs (40 GB VRAM), utilizing automatic mixed precision (AMP) to maximize computational throughput. The input volumes were cropped to a resolution of 96×96×64 and mapped to a 3-channel latent representation via an AutoencoderKL trained with composite loss weights of $w_{KL}=10^{-6},w_{p}=0.001$, and $w_{adv}=0.01$. For the generative process, we employed a DDPMScheduler with T=1000 timesteps and a scaled linear beta schedule ranging from $\beta_{\mathrm{start}}=0.0015$ to $\beta_{\mathrm{end}}=0.0195$. The Diffusion U-Net was configured with feature channels of [32, 64, 64] and attention mechanisms at the two deepest levels; it was optimized using the Adam optimizer with a learning rate of $1\times 10^{-4}$ for 150 epochs, following a 5-epoch warmup for the autoencoder. Table 1 presented the key architecture and training hyperparameters of the proposed 3DLDM model.

### 3.6. Evaluation Metrics

All quantitative evaluation was performed on the held-out test set within the lung-to-head field-of-view to align with our intended use-case (head–neck and thoracic oncology) and to avoid bias from excluded abdominal/pelvic regions. Let y denote the ground-truth PET volume in SUV units and $\hat{y}$ the synthetic PET output. Metrics were computed within a clinically relevant evaluation mask B defined on the cropped volume. Specifically, B corresponds to the foreground/body mask restricted to the lung-to-head ROI, which excludes air/background voxels and ensures that comparisons reflect uptake-relevant tissue regions rather than empty space. All reported scores are averaged across subjects in the test cohort for the global metrics.

#### 3.6.1. Global SUV-Space Fidelity (Voxel-Level)

MAE quantifies the average absolute uptake deviation between synthetic and reference PET within the evaluation mask:

(8) $$MAE(y,\hat{y})=\frac{1}{\left|B\right|}\sum_{i\in B}\left|y_{i}-{\hat{y}}_{i}\right|.$$

Here, $y_{i}$ and ${\hat{y}}_{i}$ are voxel intensities (SUV) at voxel i. Lower MAE indicates closer SUV-level agreement, reflecting improved quantitative fidelity.

We compute the peak signal-to-noise ratio (PSNR) within B using the SUV dynamic range after clipping. Let Q be the maximum possible SUV range used in evaluation (e.g., $Q=s_{max}-s_{min}$ for clipped SUV). Then:

(9) $$PSNR(y,\hat{y})=10{log}_{10}\left(\frac{Q^{2}}{\frac{1}{\left|B\right|}\sum_{i\in B}{\left(y,\hat{y}\right)}^{2}}\right)$$

A higher PSNR indicates lower reconstruction error relative to the SUV dynamic range Q, implying better preservation of uptake contrast and reduced noise-like deviations.

SSIM evaluates local structural and contrast similarity between y and $\hat{y}$ using a 3D neighborhood $\Omega_{i}$ centered at voxel i (e.g., 7×7×7). For each voxel i∈B, SSIM is computed as:

(10) $${SSIM}_{i}(y,\hat{y})=\frac{\left(2\mu_{y,i}\mu_{\hat{y},i}+c_{1}\right)\left(2\sigma_{y\hat{y},i}+c_{2}\right)}{\left(\mu_{y,i}^{2}+\mu_{\hat{y},i}^{2}+c_{1}\right)\left(\sigma_{y,i}^{2}+\sigma_{\hat{y},i}^{2}+c_{2}\right)}$$

Here, $\mu_{y,i}$ and $\mu_{\hat{y},i}$ are the local means of the ground-truth PET y and synthetic PET $\hat{y}$ within $\Omega_{ii};\sigma_{y,i}^{2}$ and $\sigma_{\hat{y},i}^{2}$ are the corresponding local variances; and $\sigma_{y\hat{y},i}$ is the local covariance between y and $\hat{y}$ in $\Omega_{i}$. The stabilizing constants are defined using the SUV dynamic range Q:

(11) $$c_{1}={\left(k_{1}Q\right)}^{2},c_{2}={\left(k_{2}Q\right)}^{2},k_{1}=0.01,k_{2}=0.03.$$

These SUV-based MAE, PSNR, and 3D SSIM metrics provide a complementary assessment of synthesis quality, capturing both voxel-wise uptake accuracy and preservation of local structural patterns. This combination offers a robust evaluation of how faithfully the generated PET reflects clinically relevant distributions within the lung-to-head region.

#### 3.6.2. Lesion-Level Detection/Localization Behavior

To complement global voxel-wise metrics, we also assessed lesion-level detection and localization to evaluate model behavior in clinically relevant focal uptake regions. Lesion-level analysis was performed using the reference lesion masks provided in the autoPET dataset. Let $G={\left\{G_{j}\right\}}_{j=1}^{NG}$ denote the set of ground-truth lesions (connected components from reference lesion masks) and $P={\left\{P_{k}\right\}}_{k=1}^{N_{P}}$ the set of predicted lesions extracted from $\hat{y}$ using the same lesion definition procedure. A predicted lesion $P_{k}$ is considered a true positive (TP) if it matches at least one ground-truth lesion $G_{j}$ under an overlap criterion; otherwise it is a false positive (FP). Any ground-truth lesion without a match is a false negative (FN). We report lesion precision and lesion recall (sensitivity) as:

(12) $$\mathrm{Precision}=\frac{TP}{TP+FP},\mathrm{Recall}=\frac{TP}{TP+FN}$$

We also report the number of detected lesions ($N_{P}$) and the number of false positive lesions (*FP*) per scan as complementary indicators of model behavior.

(13) $$FP\_\mathrm{rate}=\frac{1}{N}\sum_{j=1}^{N}Count\left(FP_{j}\right)$$

where *N* is the total number of subjects in the test cohort. This metric is a primary indicator of clinical “hallucination” risk.

#### 3.6.3. Lesion-Level SUV Quantification Accuracy

To quantify uptake fidelity specifically within lesions, we compute SUV errors restricted to lesion voxels. Let $L=\overset{N_{G}}{\underset{j=1}{⋃}}G_{j}$ be the union of ground-truth lesion voxels (within the lung-to-head ROI). Lesion SUV MSE is:

(14) $${MSE}_{\mathrm{les}}\left(y,\hat{y}\right)=\frac{1}{\left|L\right|}\sum_{i\in L}{\left(y_{i}-{\hat{y}}_{i}\right)}^{2}.$$

We also report lesion NMSE (%) to normalize by lesion uptake magnitude:

(15) $${NMSE}_{\mathrm{les}}(y,\hat{y})=\frac{\sum_{i\in L}{\left(y_{i}-{\hat{y}}_{i}\right)}^{2}}{\sum_{i\in L}y_{i}^{2}},{NMSE}_{\mathrm{les}}(\%)=100\times {NMSE}_{\mathrm{les}}(y,\hat{y}).$$

These lesion-restricted measures emphasize quantitative fidelity where PET is clinically interpreted most frequently (hypermetabolic foci) and complement global ROI-based metrics that may dilute small but important regions.

#### 3.6.4. Volume Agreement

Finally, we evaluate whether synthesized PET preserves lesion extent patterns by comparing lesion volumes derived from lesion masks. Let $v_{j}$ be the ground-truth volume of lesion $G_{j}$ (in ${mm}^{3}$, computed from voxel count times voxel volume) while ${\hat{v}}_{j}$ is the matched predicted lesion volume for the same lesion (using the TP matching). The lesion volume MAPE (%) is:

(16) $${MAPE}_{v}(\%)=\frac{100}{N_{TP}}\sum_{j\in M}\frac{\left|v_{j}-{\hat{v}}_{j}\right|}{v_{j}+\epsilon },$$

where M indexes matched TP lesions, $N_{TP}=|M|$, and ϵ is a small constant to avoid division by zero (e.g., $\epsilon =10^{-6})$. We also report lesion volume correlation using Pearson’s r between matched lesion volumes:

(17) $$r_{v}=\frac{\sum_{j\in M}\left(v_{j}-\bar{v}\right)\left({\hat{v}}_{j}-\bar{\hat{v}}\right)}{\sqrt{\sum_{j\in M}{\left(v_{j}-\bar{v}\right)}^{2}}\sqrt{\sum_{j\in M}{\left({\hat{v}}_{j}-\bar{\hat{v}}\right)}^{2}}},$$

where $\bar{v}$ and $\bar{\hat{v}}$ are means over matched lesions. Higher $r_{v}$ and lower ${MAPE}_{v}$ indicate better agreement in lesion extent.

To this end, combining global SUV-space fidelity metrics (MAE, PSNR, SSIM) with lesion-level detection behavior (precision, recall, and false-positive counts) and lesion-specific quantification and extent measures (lesion SUV MSE, lesion NMSE%, lesion volume MAPE, and lesion volume correlation) provides a comprehensive evaluation of synthesis quality, capturing both cohort-level uptake fidelity and performance in clinically salient focal uptake regions within the lung-to-head field-of-view. Statistical comparisons between 3D-LDM and each baseline were performed at the subject level using paired measurements from the same held-out test cases. Because normality of subject-level metric distributions could not be assumed, we used two-sided Wilcoxon signed-rank tests for paired comparisons. *p*-values therefore represent paired subject-level differences between each baseline and 3D-LDM, rather than voxel-level comparisons.

## 4. Results

In this section, we present a comprehensive evaluation of the proposed 3D latent diffusion model (3D-LDM) for cross-modality CT-to-PET synthesis. To rigorously assess the clinical viability and generation fidelity of our framework, we employ a multi-faceted validation strategy comparing 3D-LDM against four state-of-the-art baselines, including GAN-based methods (CycleGAN, Pix2Pix) and transformer/CNN-based architectures (SwinUNeTr, nnU-Net). Our analysis begins with a qualitative visual assessment of tracer uptake recovery and anatomical preservation (Section 4.1), followed by a detailed quantitative benchmarking using standard similarity metrics (MAE, PSNR, SSIM) and statistical significance testing (Section 4.2). We then examine the model’s stability (Section 4.3) and error distribution through correlation analysis (Section 4.4). Furthermore, to evaluate the model’s performance on clinically critical features, we conduct a specialized analysis of lesion detection and localization accuracy (Section 4.5), reporting precision, recall, and false-positive rates. We then assess the model’s quantitative reliability through lesion-level quantification accuracy and SUV fidelity (Section 4.6). Finally, we benchmark the model complexity and inference time (Section 4.7), demonstrating that our 3D-LDM achieves superior robustness and high-fidelity functional imaging while remaining computationally efficient.

### 4.1. Overall Quantitative Performance on the Test Set

Table 2 summarizes the quantitative comparison of CT-to-PET synthesis methods on the held-out test cohort (*n* = 150) within the lung-to-head (head–neck and thoracic) region, evaluated in SUV space using MAE, PSNR, and SSIM. Across all three metrics, 3D-LDM (Ours) achieves the strongest overall performance, indicating a more faithful reconstruction of PET uptake patterns while simultaneously preserving structural similarity. Specifically, our method attains the lowest MAE of 303.05 ± 22.16 (95% CI: 299.48–306.63), outperforming Pix2Pix 336.65 ± 14.85 (95% CI: 334.26–339.05) and CycleGAN 339.64 ± 19.68 (95% CI: 336.47–342.82), and further improving over nnU-Net 356.34 ± 23.63 (95% CI: 352.53–360.15) and SwinUNETR 372.43 ± 11.41 (95% CI: 370.59–374.27). These reductions in MAE reflect a consistent decrease in voxel-wise SUV deviation, suggesting that the proposed latent diffusion formulation better captures the intensity distribution of PET uptake compared with adversarial (CycleGAN/Pix2Pix) and purely discriminative synthesis baselines (nnU-Net/SwinUNETR).

Beyond error magnitude, the proposed method also provides superior signal and structural fidelity. 3D-LDM yields the highest PSNR of 32.64 ± 1.79 (95% CI: 32.35–32.93) compared with CycleGAN 29.92 ± 1.72 (95% CI: 29.63–30.19) and Pix2Pix 27.74 ± 1.92 (95% CI: 27.43–28.05), with larger margins over SwinUNETR 20.57 ± 2.22 (95% CI: 20.21–20.93) and nnU-Net 16.57 ± 1.77 (95% CI: 16.28–16.85). A similar pattern is observed for SSIM, where 3D-LDM achieves 0.86 ± 0.03 (95% CI: 0.855–0.864), exceeding CycleGAN 0.84 ± 0.03 (95% CI: 0.835–0.844), SwinUNETR 0.83 ± 0.05 (95% CI: 0.826–0.841), Pix2Pix 0.82 ± 0.04 (95% CI: 0.813–0.826), and nnU-Net 0.79 ± 0.03 (95% CI: 0.784–0.794). Importantly, the improvements over all baselines are statistically significant across metrics, with extremely small *p*-values (e.g., down to $10^{-96}$), supporting the theory that the observed gains are unlikely to be explained by random variation in the test cohort. Paired subject-level comparisons showed statistically significant differences between 3D-LDM and all baselines across MAE, PSNR, and SSIM using two-sided Wilcoxon signed-rank tests, with all comparisons remaining significant at *p* < 0.001.

To sum up, these results indicate that the proposed conditional 3D latent diffusion approach not only reduces SUV reconstruction error but also produces PET volumes with higher perceptual and structural consistency, providing a more reliable synthesis across the lung-to-head anatomical region than transformer-based, CNN-based, and GAN-based alternatives.

### 4.2. Performance Distribution and Robustness

**Figure 3:**
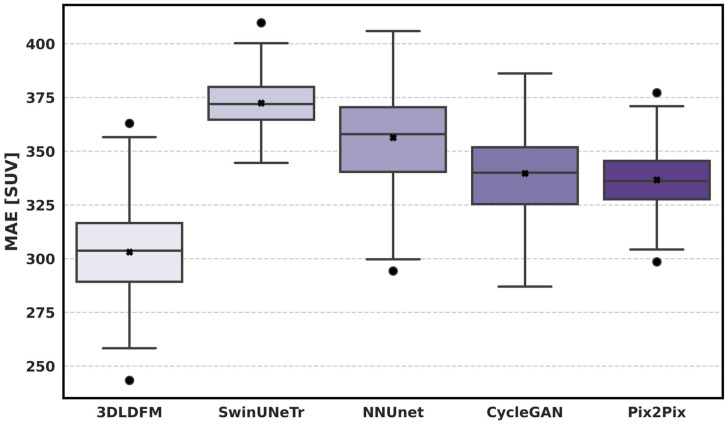
Distribution of SUV-based mean absolute error across the held-out test set for CT-to-PET synthesis.

While Table 2 reports average performance, robustness requires assessing how consistently each method performs across individual test subjects. Figure 3 presents the distribution of MAE (SUV) across the held-out test set for all methods, where lower values indicate better uptake reconstruction. The proposed 3D-LDM shows the most favorable distribution, with the lowest central tendency (median/mean marker) and a comparatively compact interquartile range, indicating not only improved average accuracy but also more stable performance across patients. In contrast, SwinUNETR exhibits the highest MAE distribution (shifted upward), suggesting systematic difficulty in recovering SUV fidelity despite its strong representational capacity. nnU-Net also demonstrates elevated MAE with broader variability, implying reduced consistency across cases. Among GAN-based baselines, CycleGAN and Pix2Pix achieve lower MAE than the CNN/transformer baselines but still remain clearly above 3D-LDM, with wider spread and outliers that reflect occasional large reconstruction deviations. Overall, the MAE distribution analysis supports that the proposed latent diffusion formulation delivers a consistent reduction in voxel-wise SUV error across the cohort, rather than relying on a small subset of favorable cases.

**Figure 4:**
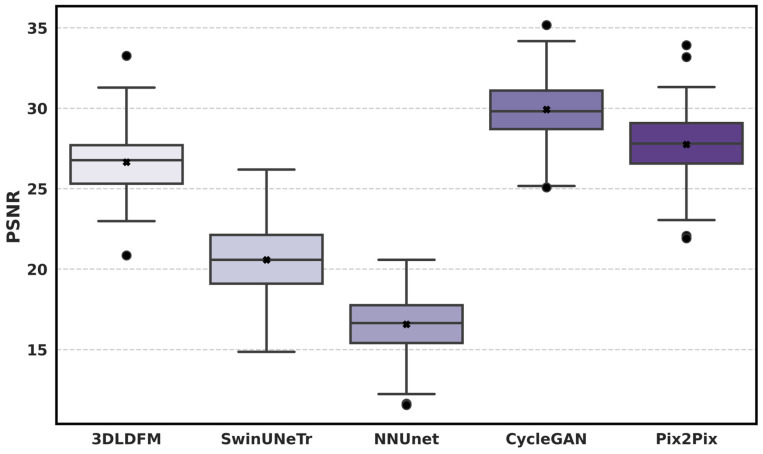
Distribution of PSNR across the held-out test set for CT-to-PET synthesis. Boxplots summarize subject-level PSNR for 3D-LDM (ours) and baseline methods (SwinUNETR, nnU-Net, CycleGAN, Pix2Pix) within the lung-to-head (head–neck and thoracic) region.

Figure 4 shows the subject-level distribution of PSNR for each CT-to-PET synthesis method within the lung-to-head (head–neck and thoracic) region, where higher values indicate better signal fidelity and lower reconstruction noise relative to the SUV dynamic range. Among all methods, 3D-LDM demonstrates the strongest overall PSNR distribution, with a high central tendency and relatively limited spread across test subjects. CycleGAN follows as the strongest baseline under this metric, whereas Pix2Pix shows intermediate performance and broader variability. In contrast, SwinUNETR and nnU-Net demonstrate substantially lower PSNR values, implying less faithful recovery of PET intensity patterns and greater distortion in the synthesized volumes. Pix2Pix achieves intermediate-to-high PSNR but shows broader variability and more pronounced outliers than 3D-LDM, consistent with occasional failure cases. Overall, the PSNR distribution analysis highlights that while some adversarial approaches can yield high global signal fidelity, 3D-LDM offers a strong balance of high PSNR and stability across subjects, which is essential for reliable CT-to-PET synthesis at cohort scale.

**Figure 5:**
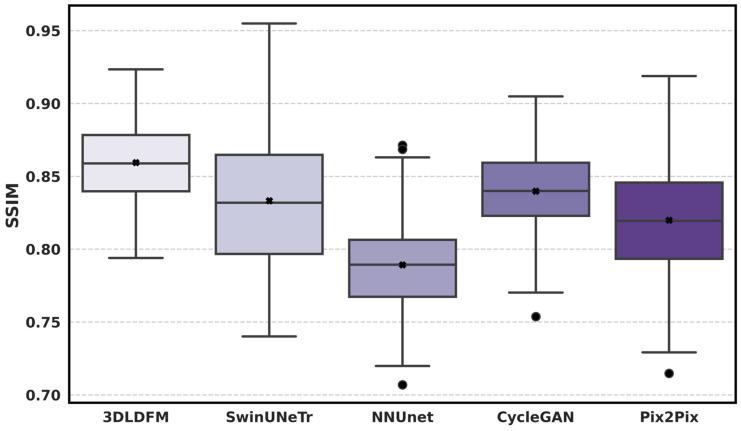
Distribution of SSIM across the held-out test set for CT-to-PET synthesis.

Figure 5 reports the subject-level distribution of SSIM for each CT-to-PET synthesis method within the lung-to-head region, where higher SSIM indicates better preservation of local structural patterns and contrast relationships in the synthesized PET relative to the reference. 3D-LDM (ours) achieves the strongest overall SSIM profile, with the highest central tendency and a tight interquartile range, indicating that the proposed latent diffusion approach preserves structural uptake patterns consistently across patients. CycleGAN follows closely, suggesting that adversarial training can recover a visually coherent structure, but its distribution remains slightly shifted downward compared with 3D-LDM and shows broader variability in some cases. In contrast, nnU-Net exhibits the lowest SSIM values, reflecting weaker structural similarity and reduced anatomical-uptake consistency. SwinUNETR and Pix2Pix show intermediate SSIM performance, but both display wider spreads and more pronounced low-end tails, consistent with occasional cases where structural fidelity degrades. Overall, SSIM distributions reinforce that 3D-LDM provides more reliable structural preservation in synthetic PET, complementing the SUV error metrics and supporting its suitability for robust cohort-scale CT-to-PET translation.

### 4.3. Metric Relationships and Trade-Offs

**Figure 6:**
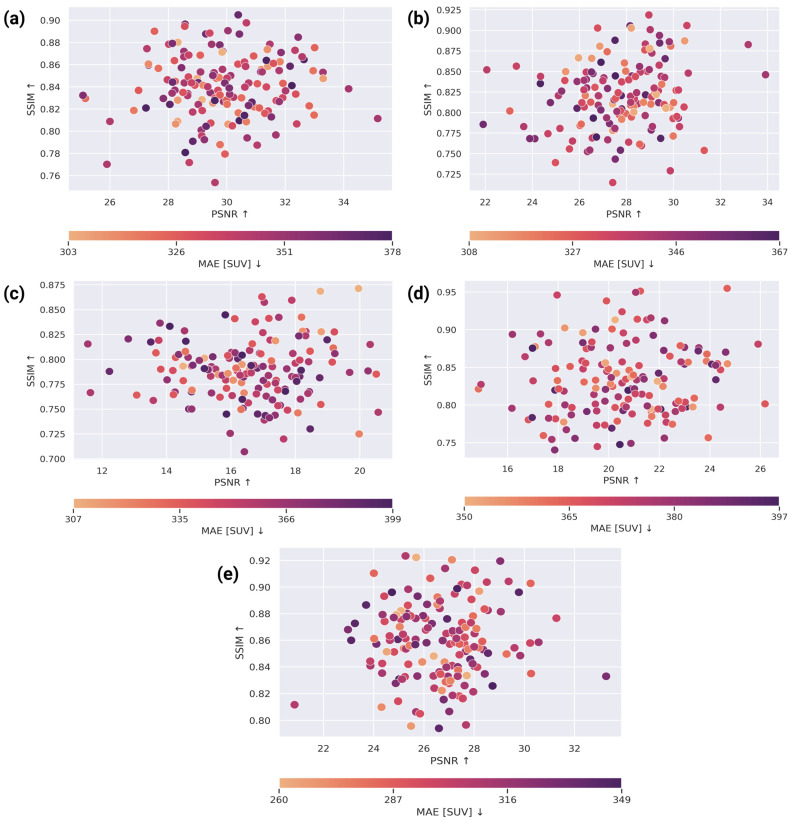
Cohort-level relationship between signal fidelity and structural similarity for CT-to-PET synthesis methods. Scatter plots show PSNR (*x*-axis, ↑) versus SSIM (*y*-axis, ↑) across test subjects, with point color indicating MAE in SUV (↓). Panels correspond to (**a**) CycleGAN; (**b**) Pix2Pix; (**c**) nnU-Net; (**d**) SwinUNETR; and (**e**) 3D-LDM (ours). The proposed 3D-LDM demonstrates tighter high-SSIM distribution with consistently lower MAE, supporting improved uptake fidelity and structural preservation across subjects. Warmer/lighter colors denote lower MAE (smaller SUV error), whereas darker colors indicate higher MAE and larger uptake deviations.

To better understand how synthesis quality varies across subjects and to characterize trade-offs between complementary fidelity measures, we analyzed the joint relationship between PSNR (signal fidelity, ↑) and SSIM (structural similarity, ↑) while encoding MAE in SUV (voxel-wise uptake error, ↓) as the point color (Figure 6). This visualization is clinically informative because an ideal CT-to-PET synthesis method should simultaneously (i) reduce SUV deviation (low MAE); (ii) maintain global signal fidelity (high PSNR); and (iii) preserve local uptake structure (high SSIM), particularly within the lung-to-head region where tumor burden and physiologic uptake patterns coexist.

Across all methods, PSNR and SSIM show a generally positive association, reflecting that improvements in global fidelity often co-occur with improved structural similarity. However, the dispersion and color gradients reveal method-specific behavior and stability. CycleGAN (Figure 6a) and Pix2Pix (Figure 6b) concentrate on many cases at relatively high PSNR and moderate-to-high SSIM, indicating that adversarial objectives can yield strong global agreement under PSNR. Nevertheless, both methods exhibit broader spread and more frequent color shifts toward higher MAE for subsets of cases, suggesting that high PSNR does not necessarily guarantee low SUV error and that uptake deviations can persist despite visually plausible structure—an important consideration for clinical interpretation where absolute uptake accuracy matters. nnU-Net (Figure 6c) shows a pronounced shift toward lower PSNR and lower SSIM with many higher-MAE points, consistent with reduced fidelity in both global intensity reconstruction and structural pattern preservation. SwinUNETR (Figure 6d) demonstrates moderate clustering but remains shifted toward lower PSNR compared with adversarial methods and 3D-LDM, with a wider spread in SSIM that indicates variable structural preservation across subjects.

In contrast, 3D-LDM (ours; Figure 6e) exhibits the most favorable joint distribution, with a dense cluster in the high-SSIM regime and PSNR values that remain consistently competitive across the cohort. Importantly, the MAE color distribution in our panel is shifted toward lower error values, indicating that improvements are not limited to a single metric but reflect a coordinated gain in uptake accuracy and structural fidelity. Clinically, this pattern suggests that the proposed conditional latent diffusion model better controls the common failure mode where a method produces structurally plausible PET but with biased or inconsistent SUV magnitudes. Lastly, the metric relationship analysis supports that 3D-LDM provides a more reliable balance between quantitative uptake correctness (MAE) and perceptual/structural realism (PSNR/SSIM), which is essential for robust CT-to-PET synthesis intended for downstream quantitative assessment and visualization in the lung-to-head oncology setting.

### 4.4. Representative Case Evaluation with Error Map Interpretation

**Figure 7:**
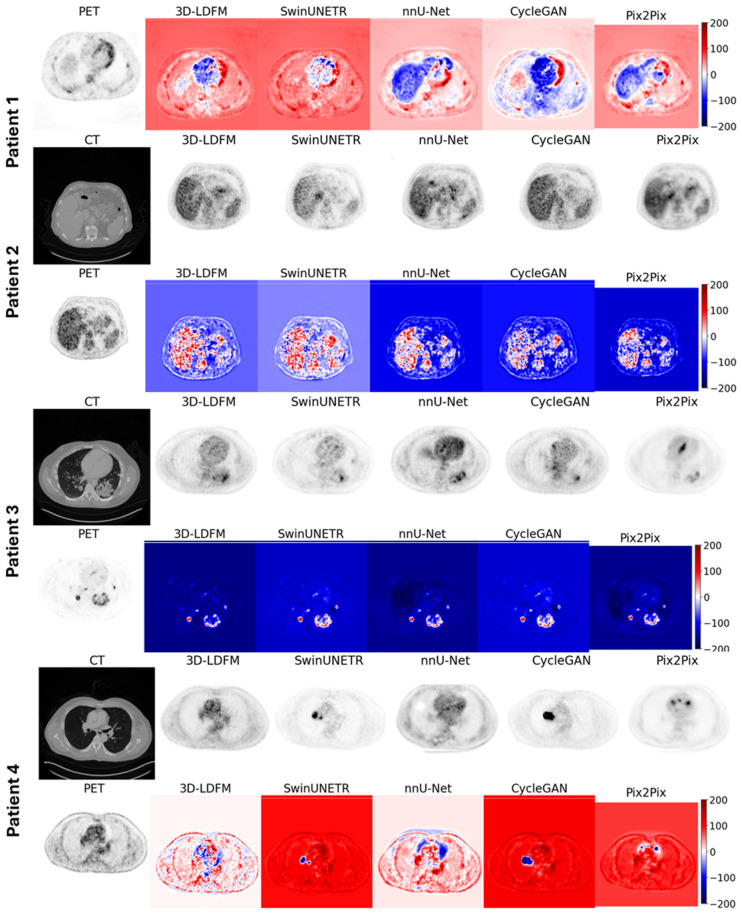
Qualitative comparison of synthetic PET generation in the lung-to-head region. Representative test cases are shown with the input CT, reference PET, and synthetic PET outputs from each method.

Figure 7 provides a case-based qualitative comparison of CT-to-PET synthesis across four representative test subjects in the lung-to-head region, combining reference PET, input CT, and method-specific reconstructions to reveal clinically meaningful error signatures that are not fully captured by summary statistics alone. For each patient, the synthesized PET volumes from 3D-LDM and the competing baselines are shown alongside voxel-wise error maps (synthetic-reference PET) using a diverging scale, where red indicates uptake overestimation and blue indicates underestimation. This visualization is particularly important in oncologic PET because clinically relevant interpretation depends not only on structural plausibility, but also on correct localization, contrast, and magnitude of uptake patterns, especially near lesions and physiological hotspots.

Across the displayed cases, 3D-LDM consistently produces reconstructions that better preserve the spatial distribution of uptake while maintaining tighter error patterns, with errors tending to remain localized rather than spreading across large anatomical regions. In contrast, the baseline methods exhibit more pronounced and structured failure modes. The transformer/CNN baselines (SwinUNETR and nnU-Net) show evidence of over-smoothing and regional bias, where uptake appears blurred or redistributed, leading to broader areas of systematic under- or overestimation on the difference maps. The GAN-based approaches (CycleGAN and Pix2Pix) frequently generate visually plausible uptake textures; however, the error maps reveal that these models can introduce inconsistent intensity scaling and patchy residuals, reflecting cases where the synthetic PET looks reasonable at a glance yet still deviates substantially from the reference uptake magnitude. Importantly, the error maps highlight that some methods can produce large-magnitude signed errors over extended regions, which is clinically undesirable because it may distort quantitative uptake assessment and potentially alter lesion conspicuity.

Finally, these representative cases reinforce the quantitative findings by demonstrating that the proposed latent diffusion formulation better controls both global uptake fidelity and local structural consistency, yielding synthetic PET volumes with fewer large, structured residuals and more reliable uptake reconstruction across heterogeneous patient presentations.

### 4.5. Lesion Detection and Localization Accuracy

In clinical PET imaging, the accurate synthesis of focal uptake is more critical than global structural similarity, as missed lesions or false positives directly impact diagnostic reliability. Using a lesion-level evaluation on the lung-to-head (HN-TH) test cohort, we report detection and localization performance via the number of detected lesions, false positives, and classification-style metrics including precision and recall (Table 3). The proposed 3D-LDM demonstrates robust lesion detection capabilities, successfully identifying an average of 3.19 lesions per scan (95% CI: 2.78, 3.59). This performance closely matches that of the much larger, segmentation-optimized nnU-Net (3.41, 95% CI: 2.95, 3.88). Notably, our model maintains a competitive precision of 0.764 (95% CI: 0.71, 0.81) and a recall of 0.762 (95% CI: 0.72, 0.80). These results indicate that the latent diffusion process effectively captures the spatial distribution of tracer uptake without introducing excessive false-positive artifacts, which were kept to a low average of 0.72 per scan (95% CI: 0.59, 0.85). In contrast, the Pix2Pix baseline exhibited a higher false-positive rate of 0.81 (95% CI: 0.65, 0.96) and lower precision, suggesting that the stochastic nature of our diffusion approach provides a more stable and reliable metabolic reconstruction than standard adversarial methods. By operating in a compressed latent space, the 3D-LDM achieves this high-fidelity localization while maintaining a significantly smaller footprint (5.27M parameters) than transformer-based alternatives. These lesion-level findings support the feasibility of synthesizing focal uptake patterns but should be interpreted cautiously. A recall of approximately 0.76 indicates that some reference lesions may be missed, while an average of 0.72 false-positive lesions per scan could affect interpretation if synthetic PET were used diagnostically. Therefore, the current model is best positioned as a decision-support or research tool rather than a standalone lesion-detection system. Clinical translation will require prospective validation, reader-in-the-loop assessment, conservative operating thresholds, and uncertainty-based flagging of unreliable regions.

### 4.6. Lesion-Level Quantification Accuracy and SUV Fidelity

To assess quantitative reliability at clinically relevant uptake sites, we evaluate lesion-wise SUV fidelity using SUV MSE (the mean squared error of SUV within lesions) and lesion NMSE (%) (uptake error normalized to account for lesion intensity scale), complemented by lesion extent agreement via volume correlation and volume MAPE (%) (Table 4). The 3D-LDM achieved the lowest Normalized Mean Square Error (NMSE) of 11.37%, significantly outperforming nnU-Net (12.67%) and SwinUNeTr (12.35%). Furthermore, our model showed the highest volume correlation (0.70) and a competitive volume MAPE of 49.05%, indicating that the morphology of synthesized lesions is more consistent with the ground truth compared to standard GAN or transformer approaches. While CycleGAN achieved a lower absolute SUV MSE, the 3D-LDM provides a more balanced profile of structural and quantitative accuracy, making it highly suitable for lesion-level analysis.

### 4.7. Model Complexity and Inference Time

**Figure 8:**
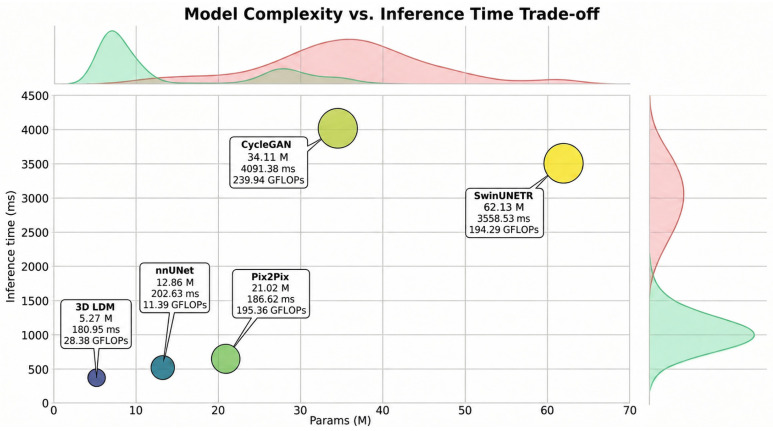
Model complexity and inference time.

To further evaluate the proposed 3D-LDM, we conducted a comparative analysis of model complexity and computational efficiency against several state-of-the-art architectures, including nnU-Net, Pix2Pix, CycleGAN, and SwinUNETR. The trade-offs between parameter count, inference latency, and computational cost (GFLOPs) are visualized in Figure 8. The findings illustrated in Figure 8 reveal that the 3D-LDM maintains a superior efficiency profile compared to established benchmarks. In terms of model compactness, the 3D-LDM utilizes only 5.27 M parameters, making it significantly more lightweight than CycleGAN (34.11 M) and SwinUNETR (62.13 M). Despite the iterative nature often associated with diffusion-based generation, our latent-space approach achieves a remarkably low inference time of 180.95 ms. This performance is not only faster than the GAN-based baselines but also slightly more efficient than the nnU-Net (202.63 ms), which is widely considered the gold standard for efficiency in medical image processing.

## 5. Discussion

This study investigates conditional 3D latent diffusion as a principled solution for CT-to-PET synthesis in the head–neck and thoracic region, where PET uptake is only partially determined by anatomy and therefore inherently ambiguous. Across a large cohort, 3D-LDM reconstructed PET with improved SUV-space fidelity and structural consistency relative to strong baselines. Distributional and case-based analyses confirm that these gains reflect stable behavior across heterogeneous patients rather than being driven by a small subset of favorable cases.

A key theoretical advantage of diffusion-based modeling is that it learns conditional distribution rather than a single deterministic mapping. CT-to-PET translation is fundamentally one-to-many: physiological and pathologic uptake depend on tracer kinetics, patient metabolism, and protocol factors such as uptake time, none of which are fully encoded in CT alone. Deterministic regressors and adversarial approaches can collapse this uncertainty into over-smoothed “average” uptake patterns, or produce visually plausible textures with systematic scaling bias and calibration drift, both of which are consequential for quantitative PET interpretation. Diffusion models, formulated as a stochastic denoising process, are naturally aligned with this uncertainty-aware setting [28,36,41,42]. In the present implementation, we generated one PET output per CT case using a fixed inference protocol to ensure standardized quantitative comparison with deterministic baselines. Therefore, the synthesized PET should be interpreted as a representative conditional synthesis from the learned CT-conditioned PET distribution, rather than a complete characterization of all plausible uptake patterns. In practical use, similar CT appearances that may correspond to malignancy, inflammation, or physiologic uptake would still require measured PET, clinical context, and physician interpretation.

Operating in latent space is especially relevant for volumetric PET synthesis. Latent diffusion retains the generative strengths of diffusion while substantially reducing the compute and memory demands that make pixel-space diffusion impractical for 3D medical volumes [26,43]. True 3D modeling additionally preserves cross-slice context and mitigates out-of-slice information loss inherent to slice-wise generation, which is important because oncologic uptake patterns frequently extend across multiple slices (e.g., nodal chains, multifocal disease) [44,45]. Within the proposed framework, three design choices contribute to these properties. First, lung mask-based cropping to the head–neck and thoracic region reduces irrelevant anatomical variability, focuses learning on clinically prioritized uptake patterns, and limits spurious correlations from anatomy outside the intended evaluation region. Second, the two-stage training strategy, in which the autoencoder learns a compact PET manifold before the diffusion model learns conditional generation within it, provides a more stable optimization path than pixel-space generation for 3D data. Third, benchmarking against transformer-, CNN-, and GAN-based baselines confirms that the observed benefit derives from the generative objective and probabilistic formulation rather than architectural scale alone, which is particularly relevant when targeting quantitative PET fidelity. Together, these choices yield a pipeline that is scalable to large datasets and adaptable to protocol changes via fine-tuning [15].

From a clinical perspective, the central question is not whether synthetic PET appears realistic, but whether it preserves clinically meaningful uptake distributions with sufficient reliability for downstream use. SUV quantification is sensitive to protocol and patient factors including uptake time, blood glucose level, and scanner calibration; consequently, scaling bias and intensity drift can materially affect staging, response assessment, and longitudinal comparison [46]. SUV-based and lesion-level detection metrics together provide complementary evidence of fidelity, while qualitative error maps reveal clinically relevant failure signatures such as structured over- or under-estimation and blurring of heterogeneous uptake. Nonetheless, synthetic PET should not substitute for measured PET in clinical decision-making without prospective validation demonstrating safety, calibration stability, and generalization across sites and protocols. Potential clinical utility should therefore be viewed as adjunctive rather than substitutive. In realistic use, CT-generated PET may support triage when PET access is limited or delayed, provide PET-like context for research or planning workflows, help prioritize patients for confirmatory PET, and serve as an additional visualization layer for multidisciplinary review. However, it should not be used for definitive staging, therapy-response assessment, radiation target definition, or treatment selection without measured PET confirmation and prospective clinical validation.

Several limitations warrant consideration. First, lesion distribution and anatomical diversity were not stratified at split time; to mitigate region-driven confounding, we enforce a consistent head–neck and thoracic field-of-view across all subjects and compute all metrics within that standardized region. Second, conditioning relies on CT alone, whereas PET uptake and SUV scaling are also influenced by covariates such as time post-injection, injected activity, blood glucose, and reconstruction settings, which may not be consistently available in retrospective datasets. Incorporating such metadata as learned embeddings injected into the generative backbone via feature-wise modulation represents a natural next step toward improved calibration and reduced protocol-dependent intensity bias [47]. Third, diffusion enables stochastic sampling and voxel-wise uncertainty quantification across multiple generations per patient, which could explicitly represent uptake ambiguity and support more conservative use in decision support; this capability was not fully exploited in the present work and warrants future investigation. Future work should explicitly generate multiple PET samples for each CT input and summarize voxel-wise variability across samples as uncertainty maps. Such maps may help identify ambiguous regions where the model is uncertain, including lesions with overlapping malignant, inflammatory, or physiologic uptake patterns. Fourth, excluding the abdomen and pelvis limits direct applicability to whole-body staging. Abdominal synthesis is particularly challenging due to high and variable physiologic FDG activity and motion effects, and we intentionally scope the present work to the head–neck and thoracic region to enable controlled volumetric evaluation, while recognizing whole-body synthesis as an important future direction requiring region-aware strategies and additional validation. Fifth, a formal ablation isolating the contributions of latent-space versus pixel-space diffusion and 3D versus 2D generation would further strengthen causal attribution. Pixel-space diffusion at volumetric resolution was computationally infeasible within the available budget; however, prior work provides strong evidence that latent diffusion reduces compute while preserving synthesis quality, and that volumetric modeling avoids the out-of-slice information loss inherent to 2D synthesis.

## 6. Conclusions

In this work, we presented a conditional 3D latent diffusion framework (3D-LDM) for CT-to-PET synthesis within the lung-to-head (head–neck and thoracic) region, leveraging a large, paired cohort of 900 patients with a fixed 600/150/150 train/validation/test split. By performing diffusion in a compact latent space, the proposed approach enables efficient volumetric generation while preserving 3D contextual information critical for anatomically coherent uptake reconstruction. Quantitative evaluation in SUV space demonstrates that 3D-LDM achieves superior overall performance relative to strong transformer-, CNN-, and GAN-based baselines, with statistically significant improvements across MAE, PSNR, and SSIM. Distributional analyses further indicate that these gains are consistent across test subjects rather than driven by a small subset of favorable cases, and representative case studies with error maps confirm reduced structured residuals and improved preservation of clinically relevant uptake patterns. Lastly, these findings support conditional 3D latent diffusion as a promising decision-support approach for PET-like functional synthesis from CT. However, synthetic PET should not be considered a replacement for measured diagnostic PET. Future work should focus on cross-site generalization, uncertainty estimation, incorporation of acquisition metadata, and prospective reader-centered validation to establish safety and clinical utility for clearly defined use scenarios.

## Figures and Tables

**Table 1:** Key architecture and training hyperparameters of the proposed 3DLDM.

Component	Parameter	Value/Setting
Data Dimensions	Input Volume Size (Cropped)	96 × 96 × 64
	Latent Space Channels	3
Autoencoder Architecture	Feature Channels	[32, 64, 64]
	Convolutional Kernels	3 × 3 × 3
	Downsampling	Stride 2
	Activation Function	SiLU (Swish)
	Final Output Activation	None (Direct Regression)
Diffusion U-Net Architecture	Feature Channels	[32, 64, 64]
	ResNet Blocks per Level	1
	Attention Levels	[False, True, True]
Training Configuration	Optimizer	Adam
	Learning Rate	1 × 10^−4^
	Batch Size	2
	Training Epochs (Diffusion)	150
	Warmup Epochs (Autoencoder)	5
Loss Function Weights	Reconstruction Loss Type	L1
	KL Divergence Weight (*w*_KL_)	10^−6^
	Perceptual Loss Weight (*w*_p_)	0.001
	Adversarial Loss Weight (*w*_adv_)	0.01
Noise Scheduler	Scheduler Type	DDPMScheduler
	Timesteps (*T*)	1000
	Schedule Type	Scaled Linear Beta
	Beta Range (*β*_start_–*β*_end_)	0.0015–0.0195
Platform	Frameworks	Python (v.3.10), PyTorch, MONAI
	Hardware	NVIDIA A100 GPU (40 GB VRAM)
	Precision	Automatic Mixed Precision (AMP)

**Table 2:** Quantitative comparison of CT-to-PET synthesis methods on the held-out test set (*n* = 150) within the lung-to-head region, reported in SUV space.

Model	MAE ↓ (Mean ± SD [95% CI])	*p*-Value	PSNR ↑ (Mean ± SD [95% CI])	*p*-Value	SSIM ↑ (Mean ± SD [95% CI])	*p*-Value
CycleGAN	339.64 (±19.68) [336.47, 342.82]	1.01 × 10^−32^	29.92 (±1.72) [29.63, 30.19]	1.91 × 10^−36^	0.84 (±0.03) [0.835, 0.844]	8.20 × 10^−9^
Pix2Pix	336.65 (±14.85) [334.26, 339.05]	1.60 × 10^−31^	27.74 (±1.92) [27.43, 28.05]	9.47 × 10^−7^	0.82 (±0.04) [0.813, 0.826]	1.76 × 10^−16^
NNUNet	356.34 (±23.63) [352.53, 360.15]	9.06 ×10^−46^	16.57 (±1.77) [16.28, 16.85]	2.05 × 10^−96^	0.79 (±0.03) [0.784, 0.794]	3.68 × 10^−43^
SwinUNeTr	372.43 (±11.41) [370.59, 374.27]	8.25 × 10^−70^	20.57 (±2.22) [20.21, 20.93]	3.28 × 10^−60^	0.83 (±0.05) [0.826, 0.841]	5.55 × 10^−9^
3D-LDM (Ours)	303.05 (±22.16) [299.48, 306.63]	-	28.64 (±1.79) [26.35, 26.93]	-	0.86 (±0.03) [0.855, 0.864]	-

Arrows indicate the direction of better performance: ↓ means lower values are better (error metrics such as MAE); ↑ means higher values are better (quality/similarity metrics such as PSNR and SSIM). *p*-values compare each baseline with 3D-LDM using two-sided Wilcoxon signed-rank tests on paired subject-level metrics.

**Table 3:** Lesion-level detection and localization performance for CT-to-PET synthesis methods.

Metric	3D-LDM (Ours) (Mean ± SD [95% CI])	SwinUNETR (Mean ± SD [95% CI])	nnU-Net (Mean ± SD [95% CI])	CycleGAN (Mean ± SD [95% CI])	Pix2Pix (Mean ± SD [95% CI])
Lesions Detected	3.19 (±2.50)	3.12 (±2.52)	3.41 (±2.89)	3.07 (±2.35)	3.16 (±2.61)
	[2.78, 3.59]	[2.71, 3.53]	[2.95, 3.88]	[2.69, 3.45]	[2.74, 3.58]
False Positives	0.72 (±0.83)	0.68 (±0.88)	0.65 (±0.73)	0.72 (±0.87)	0.81 (±0.98)
	[0.59, 0.85]	[0.54, 0.82]	[0.54, 0.77]	[0.58, 0.86]	[0.65, 0.96]
Precision	0.76 (±0.29)	0.78 (±0.29)	0.77 (±0.30)	0.78 (±0.27)	0.74 (±0.33)
	[0.71, 0.81]	[0.73, 0.83]	[0.72, 0.82]	[0.73, 0.82]	[0.68, 0.79]
Recall	0.76 (±0.23)	0.76 (±0.23)	0.77 (±0.21)	0.76 (±0.22)	0.78 (±0.21)
	[0.72, 0.80]	[0.72, 0.80]	[0.73, 0.81]	[0.72, 0.80]	[0.74, 0.81]

**Table 4:** Lesion-level quantification accuracy (SUV fidelity) and volumetric agreement for CT-to-PET synthesis methods on the lung-to-head test cohort.

Metric	3D-LDM (Ours) (Mean ± SD [95% CI])	SwinUNeTr (Mean ± SD [95% CI])	nnU-Net (Mean ± SD [95% CI])	CycleGAN (Mean ± SD [95% CI])	Pix2Pix (Mean ± SD [95% CI])
SUV MSE	8.10 (±12.55)	7.96 (±9.68)	11.97 (± 53.67)	6.98 (±11.78)	11.95 (±37.37)
	[5.90, 10.31]	[6.28, 9.63]	[2.55, 21.40]	[4.96, 9.01]	[5.44, 18.46]
Lesion NMSE (%)	11.37 (±9.57)	12.35 (±14.34)	12.67 (±12.34)	12.02 (±11.09)	12.32 (±10.51)
	[9.69, 13.05]	[9.86, 14.84]	[10.51, 14.84]	[10.12, 13.92]	[10.49, 14.15]
Lesion Vol. Correlation	0.70 (±0.57)	0.67 (±0.61)	0.63 (±0.60)	0.66 (±0.60)	0.62 (±0.65)
	[0.59, 0.80]	[0.55, 0.78]	[0.52, 0.75]	[0.55, 0.78]	[0.50, 0.74]
Lesion Vol. MAPE (%)	49.05 (±22.90)	49.39 (±22.48)	49.38 (±23.44)	49.47 (±23.89)	50.06 (±21.22)
	[45.03, 53.07]	[45.49, 53.29]	[45.26, 53.49]	[45.38, 53.57]	[46.37, 53.76]

Uptake error is summarized using SUV MSE and lesion NMSE (%), while lesion extent agreement is summarized using lesion volume correlation and volume MAPE (%). Values are mean (SD) [95% CI].

## Data Availability

The datasets used in this study are publicly available at the Cancer Imaging Archive (https://www.cancerimagingarchive.net/collection/fdg-pet-ct-lesions/; accessed on 25 June 2025).
